# Outcomes of cardiac resynchronization therapy in patients with atrial fibrillation accompanied by slow ventricular response

**DOI:** 10.1371/journal.pone.0210603

**Published:** 2019-01-11

**Authors:** Jin Kyung Hwang, Hye Bin Gwag, Kyoung-min Park, Young Keun On, June Soo Kim, Seung-Jung Park

**Affiliations:** 1 Division of Cardiology, Department of Medicine, Veterans Health Service Medical Center, Seoul, Korea; 2 Division of Cardiology, Department of Medicine, Samsung Medical Center, Sunkyunkwan University School of Medicine, Seoul, Korea; Temple University, UNITED STATES

## Abstract

It remains unclear as to whether cardiac resynchronization therapy (CRT) would be as effective in patients with atrial fibrillation (AF) accompanied by slow ventricular response (AF-SVR, < 60 beats/min) as in those with sinus rhythm (SR). Echocardiographic reverse remodeling was compared between AF-SVR patients (n = 17) and those with SR (n = 88) at six months and 12 months after CRT treatment. We also evaluated the changes in QRS duration; New York Heart Association (NYHA) functional class; and long-term composite clinical outcomes including cardiac death, heart transplantation, and heart failure (HF)-related hospitalization. Left ventricular pacing sites and biventricular pacing percentages were not significantly different between the AF-SVR and SR groups. However, heart rate increase after CRT was significantly greater in the AF-SVR group than in the SR group (*P* < 0.001). At six and 12 months postoperation, both groups showed a comparable improvement in NYHA class; QRS narrowing; and echocardiographic variables including left ventricular end-systolic volume, left ventricular ejection fraction, and left atrial volume index. Over the median follow-up duration of 1.6 (interquartile range: 0.8–2.2) years, no significant between-group differences were observed regarding the rates of long-term composite clinical events (35% versus 24%; hazard ratio: 1.71; 95% confidence interval: 0.23–12.48; *P* = 0.60). CRT implantation provided comparable beneficial effects for patients with AF-SVR as compared with those with SR, by correcting electrical dyssynchrony and increasing biventricular pacing rate, in terms of QRS narrowing, symptom improvement, ventricular reverse remodeling, and long-term clinical outcomes.

## Introduction

Cardiac resynchronization therapy (CRT) improves heart failure (HF) symptoms and reduces all-cause mortality in patients with a prolonged QRS duration [1]. CRT also improves quality of life and induces left ventricular (LV) reverse remodeling, especially in HF patients with sinus rhythm (SR) [2,3]. However, the majority of randomized controlled trials conducted to date only included patients with SR and excluded those with atrial fibrillation (AF). Patients with HF are more likely to develop AF as compared with the general population, and AF incidence increases with the severity of HF [4,5]. According to previous studies, up to 30% of HF patients with New York Heart Association (NYHA) functional classes III to IV develop AF [6].

Notably, the efficacy of CRT frequently declines in patients with AF [7,8] because the AF rhythm, especially when persistent or permanent, makes the restoration of atrioventricular (AV) synchrony impossible and reduces the biventricular pacing percentage (BiV-p%), compromising the inter- and intraventricular resynchronization [9,10]. Nevertheless, beneficial effect of CRT in AF patients was similar to that in those with SR when performed with AV nodal (AVN) ablation, which allows for a higher BiV-p% [10–12]. In addition, AVN ablation might be an effective treatment for tachycardia accompanied by AF patients with rapid ventricular response (RVR). The effective control of inappropriate tachycardia by CRT with AVN ablation may be one of the mechanisms making CRT as effective in AF-RVR patients as in those with SR [11,12]. However, supporting evidence for CRT efficacy in AF patients with slow ventricular response (SVR), for whom AVN ablation has no significant role for rate control, remains scarce. Therefore we sought to evaluate the efficacy of CRT in persistent/permanent AF patients with SVR versus in those with SR by comparing echocardiographic LV reverse remodeling and long-term clinical outcomes.

## Methods

### Study population

From March 2010 to December 2015, a total of 124 consecutive patients undergoing initial implantation of CRT at our institute were enrolled prospectively in the CRT registry. Of these, we excluded patients with (1) follow-up loss after CRT implantation (n = 9), (2) baseline persistent AF with ventricular rate > 60 beats per minute (bpm) (n = 5), (3) newly developed persistent AF during follow-up after CRT implantation (n = 4), and (4) failed CRT implantation (n = 1). A total of 105 patients were finally included into our analysis and divided into the following two groups based on their baseline rhythm prior to CRT implantation: the AF-SVR group (persistent AF with ventricular rate < 60 bpm, n = 17) and the SR group (n = 88). All patients had the following characteristics: LV ejection fraction (LV EF) ≤ 35% on transthoracic echocardiogram, QRS duration more than 120 ms on 12-lead electrocardiogram (ECG), and drug-refractory systolic HF with NYHA class III or ambulatory IV symptoms. The institutional review board of our institute approved this study and the requirement of written informed consent was waived.

### Baseline data and assessment

Baseline characteristics including functional status based on NYHA classification, demographics, medications, and electrocardiographic and echocardiographic variables were collected retrospectively using the dedicated CRT registry database. ECG data included QRS duration and morphology. Baseline QRS morphology was classified as either left bundle branch block (LBBB) or non-LBBB. LBBB was defined when all of the following criteria were met: (1) QRS duration ≥ 130 ms; (2) QS or rS in leads V1 and V2; and (3) mid-QRS notch or slurring in two or more contiguous leads (i.e., I, aVL, V1, V2, V5, or V6) [13]. In the pacing-dependent patients undergoing upgrade to CRT, paced-QRS complexes were used for the assessment of baseline QRS duration and morphology prior to CRT implantation. Echocardiographic parameters such as LV end-systolic volume (LVESV), LV end-diastolic volume (LVEDV), LVEF, and left atrial volume index (LAVI) were assessed by Simpson’s biplane method using commercially available equipment (Vivid 7 from GE Healthcare, Chicago, IL, USA; Acuson 512 from Siemens, Munich, Germany; or Sonos 5500 from Philips, Andover, MA, USA).

### Cardiac resynchronization therapy

CRT-pacing (CRT-P) or CRT-defibrillator (CRT-D) devices were implanted using sterile standard transvenous techniques under local anesthesia. All commercially available CRT devices for biventricular pacing were used, and the choice for a device providing defibrillation backup was made according to the physician’s discretion. The pacing sites of the right ventricular (RV) or LV segments were confirmed by left anterior oblique (LAO) and right anterior oblique (RAO) fluoroscopic images. The LV leads were preferably positioned into the anterolateral, lateral, or posterolateral LV walls in the LAO view and the basal or mild-LV segments in the RAO view, respectively. In the SR group, the CRT device was programmed in the atrial-synchronous sequential pacing mode of DDD or DDDR. In the AF-SVR group, the lower rate limit was set at 60 beats per minute under the VVIR pacing mode and the maximum rate was set around 80% of the age-predicted maximum heart rate (HR). After CRT implantation, the optimization of AV and ventriculoventricular (VV) delays was performed to achieve the greatest stroke volume or narrowest QRS duration on echocardiographic or electrocardiographic assessment during the index hospitalization.

### Follow-up and definitions of outcomes

Clinical and device follow-up was performed every three months after CRT implantation according to our center’s routine practice. Surface 12-lead ECG recordings and BiV-p% reports were taken at every clinic visit. The average BiV-p% was calculated using the values obtained during the first three visits. Adjustment of the CRT pacing parameters including lower rate limit, pacing mode, and AV or VV delay was left up to the physician’s discretion during the follow-up period. The primary outcome was echocardiographic reverse remodeling at six months and 12 months after CRT. Echocardiographic reverse remodeling was measured as relative changes in LVESV and LVEF versus the baseline values (pre-CRT − post-CRT)/pre CRT × 100%. Regarding the response rate, patients with a relative reduction in LVESV of ≥ 15% were defined as regular responders, while those with a reduction of ≥ 30% were classified as super-responders [14]. Secondary outcomes included change in NYHA class; QRS duration; LVEDV and LAVI at six months and 12 months; and long-term clinical outcomes such as all-cause death, cardiac death, heart transplantation, HF-related admission, and major adverse cardiac events (MACE) during the follow-up period. The MACE was defined as composite event of cardiac death, heart transplantation, or HF-related admission. All deaths were considered to be cardiac-related deaths unless a definite noncardiac cause could be established.

### Statistical analysis

Continuous variables were presented in the format of mean ± standard deviation and differences were assessed using an independent *t*-test or the Wilcoxon rank-sum test. Categorical variables were described as a number (*n*) with a percentage (%) and differences were analyzed by Pearson’s chi-squared or Fisher’s exact test. The Cox proportional hazards model was used to compare the risks of clinical outcomes between the two groups. We considered the following covariates in the multivariate analysis that were potential confounders: age, sex, NYHA functional class, availability of back-up defibrillation with implanted device, LVEF, change in HR, and QRS duration. Event-free survival analysis for MACE was performed by means of the Kaplan–Meier method, and survival curves were compared by using the log-rank test. Longitudinal changes in LVESV, LVEF, LAVI, and QRS duration between the two groups were compared using generalized linear regression models. All tests were two-tailed and *P* < 0.05 was considered to be statistically significant. All analyses were performed using the Statistical Package for the Social Sciences version 20 for Windows software (IBM Corp., Armonk, NY, USA).

## Results

### Baseline characteristics

Baseline characteristics including proportion of male gender and ischemic etiology of LV systolic dysfunction, severity in HF symptoms, and N-terminal prohormone of brain natriuretic peptide level were not significantly different between the two groups (Table 1). In addition, there were no significant differences in QRS duration; rate of LBBB morphology; LVEF; and baseline medications including β-blockers, renin–angiotensin–aldosterone system blockers, and diuretics between the two groups. BiV-p% and the distribution of LV pacing sites in the AF-SVR group were not significantly different as compared with those of the SR group. However, baseline HR in the AF-SVR group was measured as 41 ± 8 bpm, which was much slower than that of the SR group (75 ± 15 bpm; *P* < 0.001). There was also a higher rate of previous stroke in the AF-SVR group versus in the SR group, and the AF-SVR group additionally showed a greater LA size and more prevalent tricuspid regurgitation ≥ moderate degree. In contrast, LV dimensions were larger in the SR group and there was no significant difference in the severity of mitral regurgitation.

**Table 1:** Baseline characteristics.

	AF-SVR	SR	*P* value
	(n = 17)	(n = 88)	
***Demographics***			
Age (years)	61.7 ± 12.0	63.9 ± 13.2	0.30
Male gender, n (%)	10 (58.8)	54 (61.4)	0.84
BMI(kg/m^2^)	21.7 ± 3.6	22.9 ± 3.7	0.22
Hypertension, n (%)	11 (64.7)	45 (51.1)	0.31
Diabetes, n (%)	4 (23.5)	28 (31.8)	0.50
Dyslipidemia, n (%)	2 (11.8)	21 (23.9)	0.35
Chronic kidney disease, n (%)	4 (23.5)	13 (14.8)	0.47
Stroke, n (%)	4 (23.5)	6 (6.8)	0.05
Myocardial infarction, n (%)	1 (5.9)	16 (18.2)	0.30
PCI, n (%)	1 (5.9)	13 (14.8)	0.46
CABG, n (%)	1 (5.9)	11 (12.5)	0.69
ICM, n (%)	2 (11.8)	23 (26.1)	0.35
NYHA class III, n (%)	15 (88.2)	76 (86.4)	1.00
NYHA class IV, n (%)	2 (11.8)	12 (13.6)	1.00
NT-proBNP (pg/mL)	5075 ± 8920	4183 ± 5176	0.99
***Electrocardiographic data***			
QRS duration (ms)	177 ± 36	165 ± 22	0.26
LBBB, n (%)	13 (76.5)	73 (83.0)	0.50
Non-LBBB, n (%)	4 (23.5)	15 (17.0)	0.50
Heart rate (beats/minute)	41±8	75±15	<0.001
***Echocardiographic data***			
LVEF (%)	24 ± 7	23 ± 5	0.48
LVEDV (mL)	203 ± 64	260 ± 84	0.008
LVESV (mL)	162 ± 64	201 ± 67	0.05
LAVI (mL/m^2^)	82 ± 31	58 ± 22	0.001
MR ≥ moderate, n (%)	6 (35.3)	31 (35.2)	1.00
TR ≥ moderate, n (%)	6 (35.3)	10 (11.4)	0.02
***Medications***			
Beta-blocker, n (%)	8 (47.1)	60 (68.2)	0.10
ACEI/ARB, n (%)	14 (82.4)	78 (88.6)	0.44
Spironolactone, n (%)	10 (58.8)	51 (58.0)	1.00
Diuretics, n (%)	14 (82.4)	77 (87.5)	0.70
***CRT device***			
BiV pacing percentage (%)	97± 3	97 ± 7	1.00
Lateral LV pacing sites*, n (%)	16 (94.1)	87 (98.9)	0.30
Non-apical LV pacing sites*, n (%)	17 (100)	83 (94.3)	0.59
CRT-defibrillator, n (%)	15 (88.2)	84 (95.5)	0.25

Values are presented in the formats of number (%) and mean ± standard deviation.

* Lateral LV pacing sites indicates anterolateral, lateral, and posterolateral LV walls in the LAO view, whereas nonapical LV pacing sites the basal and mid-LV segments in the RAO view where the stimulating cathodes of LV leads were located. ACEI/ARB, angiotensin-converting enzyme inhibitor/aldosterone-receptor blocker; AF-SVR, atrial fibrillation accompanied by slow ventricular response; BiV, biventricular; BMI, body mass index; CABG, coronary artery bypass graft surgery; CRT, cardiac resynchronization therapy; ICM, ischemic cardiomyopathy; LAVI, left atrial volume index; LBBB, left bundle branch block; LVEDV, left ventricular end-diastolic volume; LVEF, left ventricular ejection fraction; LVESV, left ventricular end-systolic volume; MR, mitral regurgitation; NT-proBNP, N-terminal prohormone of brain natriuretic peptide; NYHA class, New York Heart Association functional classification; PCI, percutaneous coronary artery intervention; RBBB, right bundle branch block; SR, sinus rhythm; TR, tricuspid regurgitation.

### Electrocardiographic and echocardiographic outcomes

**Fig 1:**
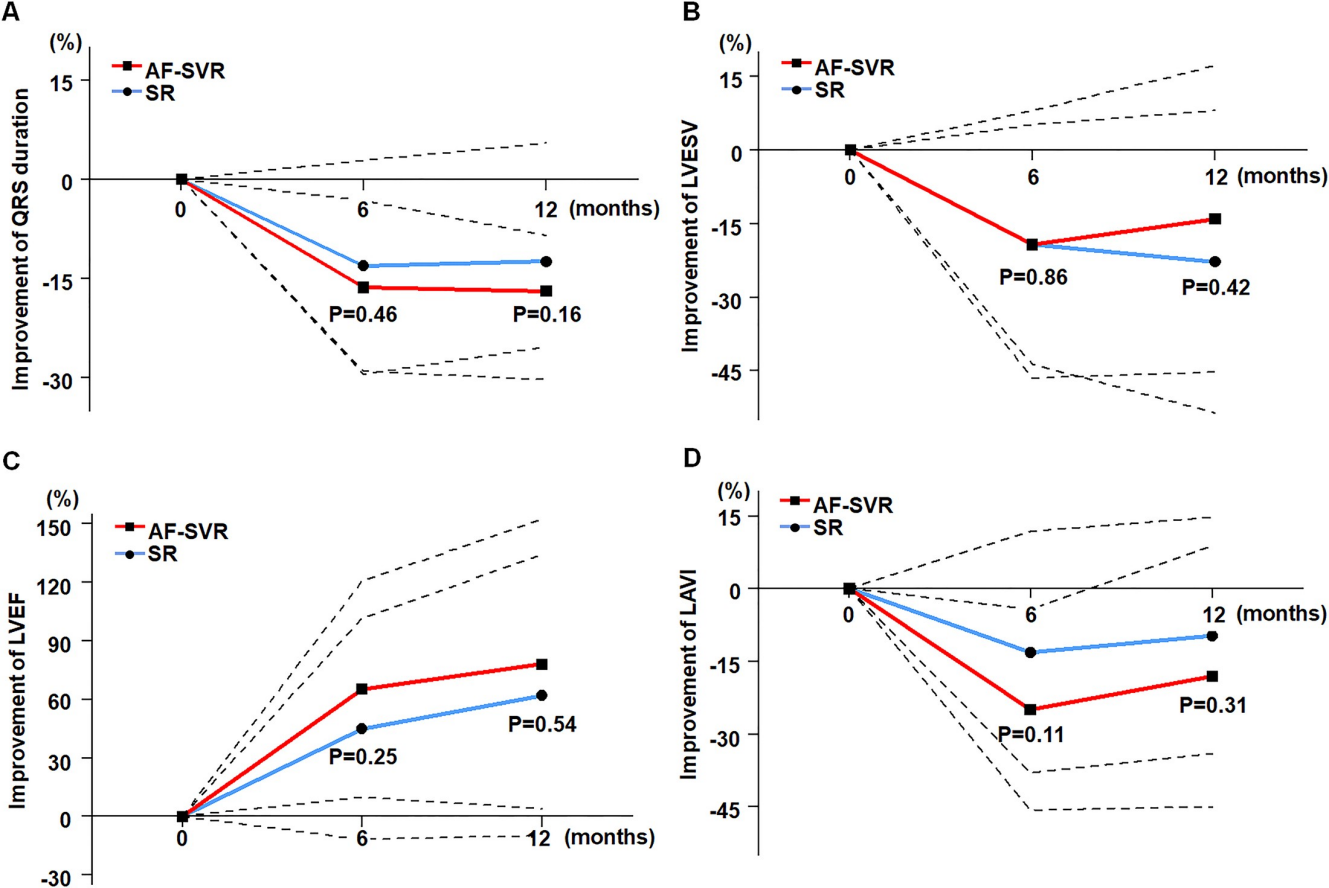
Comparable improvement of electrocardiographic and echocardiographic parameters during the first year after CRT implantation between patients with AF-SVR (red line) and patients with SR (blue line). The black dotted line indicates standard deviation. (A) Relative change in QRS duration, (B) LVESV, (C) LVEF, and (D) LAVI.

**Fig 2:**
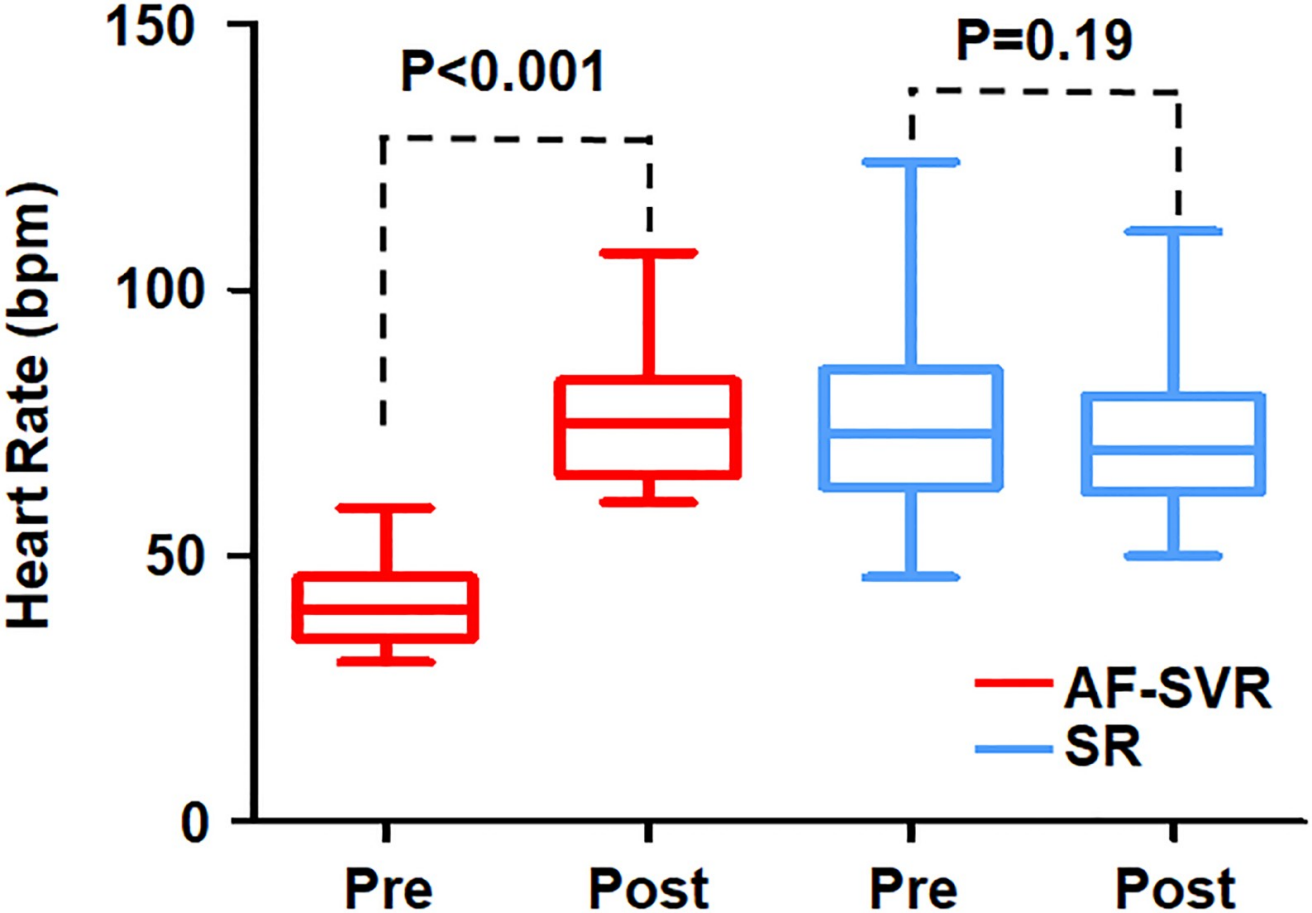
Pre- and post-CRT heart rates in each group. AF-SVR, patients with atrial fibrillation accompanied by slow ventricular response; SR, patients with sinus rhythm.

The follow-up electrocardiographic and echocardiographic assessments at six months and 12 months after CRT implantation are summarized in Table 2. There was no significant difference in the QRS duration measured at six months between the AF-SVR and SR groups (Table 2). The relative amount of QRS narrowing in the AF-SVR group was also comparable to that in the SR group (Fig 1A). However, the HR increase at six months postimplantation was prominent only in the AF-SVR group, from 40 ± 8 bpm to 75 ± 14 bpm (Fig 2). In contrast, the HR change in the SR group was negligible. So, both groups showed similar follow-up HRs (75 ± 14 bpm versus 72 ± 13 bpm; *P* = 0.44).

**Table 2:** Electrocardiographic and echocardiographic outcomes.

	AF-SVR	SR	*P* value
	(n = 17)	(n = 88)	
***Electrocardiographic outcomes***			
Sixth month			
QRS duration (ms)	147 ± 29	141 ± 22	0.44
Delta QRS duration (ms)	−30 ± 26	−24 ± 27	0.46
12^th^ month			
QRS duration (ms)	147 ± 22	142 ± 25	0.48
Delta QRS duration (ms)	−30 ± 22	−23 ± 30	0.49
***Echocardiographic outcomes***			
Sixth month			
LVESV (mL)	136 ± 56	161± 74	0.29
Delta LVESV (mL)	−26 ±46	−40 ± 54	0.26
LVESV reduction* ≥ 15%, n (%)	10 (58.8)	48 (54.5)	0.79
LVESV reduction* ≥ 30%, n (%)	6 (35.3)	27 (30.7)	1.00
LVEF (%)	35 ± 12	32 ± 12	0.22
Delta LVEF (%)	13 ± 12	9 ± 12	0.12
LAVI (mL/m^2^)	63 ± 19	48 ± 18	0.009
12^th^ month			
LVESV (mL)	135 ± 68	166 ± 88	0.31
Delta LVESV (mL)	−22 ± 50	−45 ± 64	0.19
LVESV reduction* ≥ 15%, n (%)	8 (47.0)	50 (56.8)	0.74
LVESV reduction* ≥ 30%, n (%)	5 (29.4)	40 (45.5)	0.49
LVEF (%)	40 ± 10	34 ± 13	0.10
Delta LVEF (%)	16 ± 12	12 ± 14	0.26
LAVI (mL/m^2^)	71 ± 35	50 ± 23	0.046

Values are presented in the formats of number (%) and mean ± standard deviation.

* indicates relative reduction in LVESV defined as (preCRT − postCRT)/preCRT × 100%. AF-SVR, atrial fibrillation accompanied by slow ventricular response; LAVI, left atrial volume index; LVEDV, left ventricular end-diastolic volume; LVEF, left ventricular ejection fraction; LVESV, left ventricular end-systolic volume; SR, sinus rhythm.

The amount of LV reverse remodeling estimated at six months was not significantly different between the AF-SVR and SR groups: the absolute changes in LVESV, −26 ± 46 mL versus −40 ± 54 mL, *P* = 0.26 (Table 2); the relative changes in LVESV from baseline, −19 ± 27% versus −19 ± 24%, *P* = 0.86 (Fig 1B); the regular responder rate, 58.8% versus 54.5%, *P* = 0.79; and super responder rates, 35.3% versus 30.7%, *P* = 1.00. LVEFs assessed at six months (35 ± 12% versus 32 ± 12%, *P* = 0.22) and percent change in LVEFs (65 ± 56% versus 45 ± 57%, *P* = 0.25) were comparable to each other in both groups (Fig 1C). Mean LAVI was higher in patients with AF-SVR than in those with SR at six months (*P* = 0.009) after CRT implantation, but the changes in LAVI from baseline were not significantly different between the groups (Fig 1D). The overall patterns of six-month electrocardiographic and echocardiographic changes were maintained up to the 12-month follow-up point after CRT (Fig 1).

### Change in NYHA functional status

**Fig 3:**
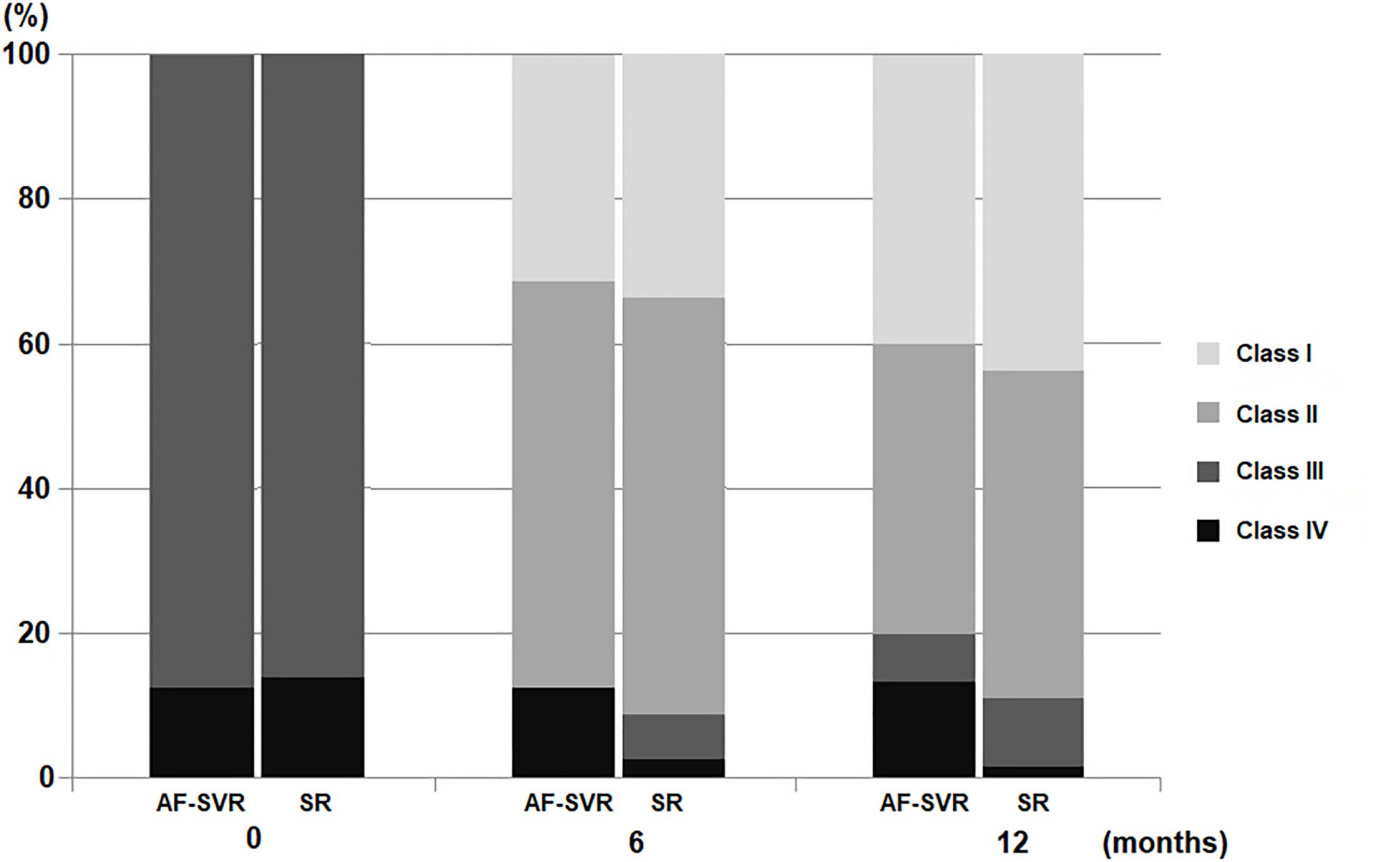
Improvement in NYHA functional class. No significant between-group differences were observed at six months and 12 months after CRT implantation. The vertical bars demonstrate the proportion of patients with a certain NYHA class in each group. AF-SVR, patients with atrial fibrillation accompanied by slow ventricular response; SR, patients with sinus rhythm.

The overall change in functional status was not significantly different between the two groups, as depicted in Fig 3. At baseline, all patients were classified as NYHA class III or ambulatory IV, but six months after CRT, 88% of patients in the AF-SVR group (n = 15) and 91% in the SR group (n = 80) had improved their NYHA functional status to class I or II (*P* = 1.00). The mean differences of NYHA functional class as compared with baseline were −1.2 ± 0.8 in the AF-SVR group and −1.3 ± 0.6 in the SR group (*P* = 0.66). The symptom improvement was well-maintained up to the 12-month follow-up visit in both groups; 82% of patients in the AF-SVR group (n = 14) and 89% in the SR group (n = 78) showed NYHA class I or II status (*P* = 0.89). The mean difference of NYHA class between at baseline versus at 12 months was −1.1 ± 0.9 versus −1.4 ± 0.7 (*P* = 0.21).

### Long-term clinical outcomes

**Fig 4:**
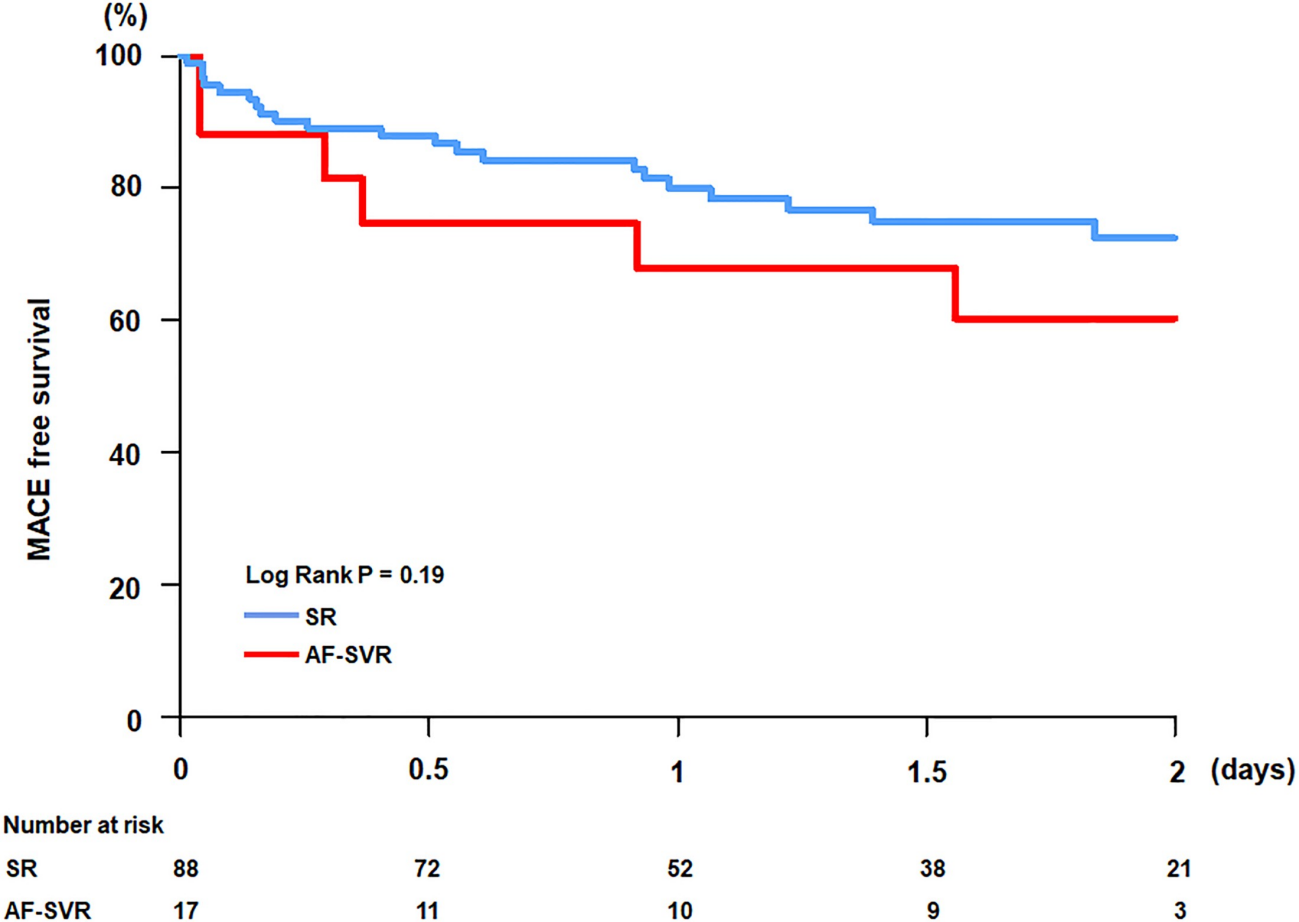
Comparison of MACE-free survival. AF-SVR, patients with atrial fibrillation accompanied by slow ventricular response; SR, patients with sinus rhythm.

During the median follow-up period of 1.6 years (interquartile range: 0.8–2.2 years), all-cause death occurred in two patients in the AF-SVR group and five patients in the SR group [11.8 versus 5.7%, adjusted hazard ratio (HR): 3.66, 95% confidence interval (CI): 0.01–12.03; *P* = 0.35] (Table 3). All death cases were turned out as cardiac deaths. The rate of admission associated with HF aggravation and the rate of heart transplantation were not significantly different between the two groups. The MACE rate was 35.3% in the AF-SVR group and 23.9% in the SR group (adjusted HR: 1.71, 95% CI: 0.23–12.48; *P* = 0.60). During the entire follow-up period, the AF-SVR and SR groups showed no significant differences in MACE-free survival rates (Fig 4).

**Table 3:** Clinical outcomes during the follow-up period.

	AF-SVR	SR	HR*(95% CI)	*P* value
	(n = 17)	(n = 88)		
All-cause death	2 (11.8)	5 (5.7)	3.66 (0.01–12.03)	0.35
Cardiac death	2 (11.8)	5 (5.7)	3.66 (0.01–12.03)	0.35
HF admission	4 (23.5)	15 (17.0)	3.17 (0.28–35.35)	0.35
Heart transplantation	2 (11.8)	6 (6.8)	4.70 (0.08–27.32)	0.46
MACE^†^	6 (35.3)	21 (23.9)	1.71 (0.23–12.48)	0.60

Values are presented in the format of number (%).

*HR was adjusted for age, sex, NYHA functional class, availability of back-up defibrillation with implanted device, LVEF, LVEDV, change in heart rate, and QRS duration.

^†^ MACE is defined as a composite event of cardiac death, heart transplantation, or admission associated with HF. AF, atrial fibrillation; AF-SVR, atrial fibrillation accompanied by slow ventricular response; CI, confidence interval; HF, heart failure; HR, hazard ratio; MACE, major adverse cardiac events; SR, sinus rhythm.

## Discussion

Atrial contraction usually accounts for 20 to 30% of cardiac output [15,16]. Therefore, the loss of atrial function caused by persistent AF can deteriorate overall cardiac performance and worsen the prognosis of HF patients. Likewise, the inability of atrial synchronous BiV pacing in HF patients with persistent AF might compromise the efficacy of CRT. Furthermore, a fast HR in AF patients could hamper effective BiV pacing by rapidly conducting intrinsic rhythms, fusion beats, and pseudofusion pacing. Indeed, a recent meta-analysis that included a total of 7,495 patients treated with CRT found higher rates of all-cause death (11 versus 7% per year; *P* < 0.001) and CRT nonresponse (35 versus 27%; *P* < 0.01) in AF patients versus in those with SR.^8^ In the RAFT trial as well, CRT was less likely to be effective in AF patients than in those with SR for preventing death or HF-related hospitalization [7].

In the present study, however, the AF-SVR group showed comparable improvement in LV reverse remodeling compared with the SR group. The rates of regular and super responders to CRT in the AF-SVR group (59% and 35%) were similar to those in the SR group (55% and 31%). In addition, no between-group differences were observed with respect to QRS narrowing, change in NYHA functional class, HF hospitalization, heart transplantation, and mortality. To our knowledge, our study is the first to show a comparable efficacy of CRT for patients with AF-SVR compared to those with SR, and our findings are consistent with the results of several large prospective CRT studies performed in chronic AF patients [9,10,17].

Several possible explanations can be offered regarding the comparable efficacy of CRT in patients with AF-SVR versus in those with SR. First, the degree of electrical resynchronization suggested by relative QRS narrowing was similar between the two groups. Next, high levels of BiV-p% were achievable in the AF-SVR group without AVN ablation due to the inherent AVN malfunction. So, the BiV-p% in the AF-SVR group was as high as that in the SR group (97 ± 3% versus 97 ± 7%, *P* = 1.00). In a recently published Cardiac Resynchronization Therapy in Atrial Fibrillation Patients Multinational Registry (CERTIFY) study, AF patients who were able to attain high levels of BiV-p% (96 ± 6%) through CRT in combination with AVN ablation showed almost equal outcomes to those obtained in patients with SR in terms of LVESV reduction, all-cause death (6.8 versus 6.1 per 100 person-years), and cardiac mortality (4.2 versus 4.0) over a median follow-up of 37 months [10]. In contrast, AF patients with lower levels of BiV-p% (87 ± 14%), which were associated with the absence of AVN ablation following CRT device implantation, showed two times higher all-cause and cardiac mortality than patients with SR. Finally, the dramatic increase in HR (from 40 ± 8 bpm to 75 ± 14 bpm), which was observed only in the AF-SVR group, might contribute to augment cardiac output (CO) and exercise tolerance in this patient group. Moreover, the increase of the BiV pacing rate in the range of 60 to 90 bpm was reportedly associated not only with CO increase but also with a decrease in filling pressures and sympathetic nerve activity [18,19].

There have not been enough data on the optimal BiV pacing rate for AF patients undergoing AVN ablation. As already mentioned above, the BiV pacing rate of 60 to 90 bpm alleviated sympathetic activity and improved hemodynamics. In a large observational study involving 488 HF patients with persistent AF, tight control of HR below 73 bpm was associated with worse survival, whereas patients with a HR of 73 to 82 bpm showed the best prognosis [20]. Interestingly, the mean value of the BiV pacing rate of the AF-SVR group (75 ± 14 bpm) fell within this range.

Taken altogether, besides correcting electrical dyssynchrony, CRT might confer the extra benefit of CO augmentation on AF patients with SVR by increasing paced HR. The relief of tachycardiomyopathy could be adjunctively obtained in AF patients with RVR by AVN ablation [11,12]. These additional benefits might make CRT as effective for AF patients as for those with SR, compensating for the AF-related lack of atrial synchronous BiV pacing.

### Limitations

There are several limitations in this study. First, the present investigation was a nonrandomized observational study. We could not control all various confounding factors that might affect results in this study. Second, the small number of patients in the AF-SVR group limits the extrapolation of our results. However, follow-up was done at a very regular interval of three months in all patients. In addition, there was no follow-up loss in the AF-SVR group. All of the nine patients who were excluded as a result of follow-up loss had SR at baseline. Moreover, consistent patterns of results were observed at 12 months as well as six months after implantation. Third, medications were prescribed at the discretion of the physician after CRT implantation, which can be another confounding factor. However, special efforts were made to give optimal medical therapy to all patients according to the latest HF guidelines.

## Conclusions

In the present investigation, the treatment efficacy of CRT was not significantly different in patients with AF-SVR as compared with in those with SR in terms of echocardiographic LV reverse remodeling, QRS narrowing, symptom improvement, and long-term clinical outcomes. The rates of regular and super responders to CRT were similar between the two groups. Our data suggest that CRT implantation provides a comparable beneficial effect in patients with AF-SVR to that achieved in patients with SR by correcting electrical dyssynchrony and increasing BiV pacing rate. Further randomized, large-scale studies are required to confirm our study results.
